# Sensitivity of Continuous Electroencephalography to Detect Ictal Activity After Cardiac Arrest

**DOI:** 10.1001/jamanetworkopen.2020.3751

**Published:** 2020-04-28

**Authors:** Jonathan Elmer, Patrick J. Coppler, Pawan Solanki, M. Brandon Westover, Aaron F. Struck, Maria E. Baldwin, Michael C. Kurz, Clifton W. Callaway

**Affiliations:** 1 Department of Emergency Medicine, University of Pittsburgh, Pittsburgh, Pennsylvania; 2 Department of Critical Care Medicine, University of Pittsburgh, Pittsburgh, Pennsylvania; 3 Department of Neurology, University of Pittsburgh, Pittsburgh, Pennsylvania; 4 Department of Neurology, Massachusetts General Hospital, Boston; 5 Department of Neurology, University of Wisconsin, Madison; 6 Department of Neurology, Pittsburgh VA Medical Center, Pittsburgh, Pennsylvania; 7 Department of Emergency Medicine, University of Alabama at Birmingham School of Medicine

## Abstract

**Importance:**

Epileptiform electroencephalographic (EEG) patterns are common after resuscitation from cardiac arrest, are associated with patient outcome, and may require treatment. It is unknown whether continuous EEG monitoring is needed to detect these patterns or if brief intermittent monitoring is sufficient. If continuous monitoring is required, the necessary duration of observation is unknown.

**Objective:**

To quantify the time-dependent sensitivity of continuous EEG for epileptiform event detection, and to compare continuous EEG to several alternative EEG-monitoring strategies for post–cardiac arrest outcome prediction.

**Design, Setting, and Participants:**

This observational cohort study was conducted in 2 academic medical centers between September 2010 and January 2018. Participants included 759 adults who were comatose after being resuscitated from cardiac arrest and who underwent 24 hours or more of EEG monitoring.

**Main Outcomes and Measures:**

Epileptiform EEG patterns associated with neurological outcome at hospital discharge, such as seizures likely to cause secondary injury.

**Results:**

Overall, 759 patients were included in the analysis; 281 (37.0%) were female, and the mean (SD) age was 58 (17) years. Epileptiform EEG activity was observed in 414 participants (54.5%), of whom only 26 (3.4%) developed potentially treatable seizures. Brief intermittent EEG had an estimated 66% (95% CI, 62%-69%) to 68% (95% CI, 66%-70%) sensitivity for detection of prognostic epileptiform events. Depending on initial continuity of the EEG background, 0 to 51 hours of monitoring were needed to achieve 95% sensitivity for the detection of prognostic epileptiform events. Brief intermittent EEG had a sensitivity of 7% (95% CI, 4%-12%) to 8% (95% CI, 4%-12%) for the detection of potentially treatable seizures, and 0 to 53 hours of continuous monitoring were needed to achieve 95% sensitivity for the detection of potentially treatable seizures. Brief intermittent EEG results yielded similar information compared with continuous EEG results when added to multivariable models predicting neurological outcome.

**Conclusions and Relevance:**

Compared with continuous EEG monitoring, brief intermittent monitoring was insensitive for detection of epileptiform events. Monitoring EEG results significantly improved multimodality prediction of neurological outcome, but continuous monitoring appeared to add little additional information compared with brief intermittent monitoring.

## Key Points

**Question:**

Does continuous electroencephalographic (EEG) monitoring improve detection of epileptiform events associated with neurological outcome or potentially treatable seizure among patients resuscitated from cardiac arrest compared with brief intermittent EEG?

**Findings:**

In this cohort study including 759 patients who were resuscitated from cardiac arrest, continuous monitoring for nearly 48 hours was needed to achieve 95% sensitivity for prognostic epileptiform events; compared with brief intermittent EEG, continuous monitoring did not improve prediction of outcome at hospital discharge. Potentially treatable seizures were uncommon and rarely detected by brief intermittent monitoring.

**Meaning:**

Compared with continuous EEG, brief intermittent EEG is insensitive for detection of potentially treatable seizures after cardiac arrest but may be sufficient to guide multimodality prediction of outcome at hospital discharge.

## Teaser

This cohort study of patients resuscitated following cardiac arrest examines whether continuous monitoring of electroencephalographic activity improves detection of epileptiform events associated with neurological outcome and potentially treatable seizures.

## Introduction

Sudden cardiac arrest affects more than 600 000 individuals in the US each year.^1^ Most patients hospitalized after resuscitation from cardiac arrest are initially comatose,^2,3^ and of these, 25% to 50% have electroencephalographic (EEG) activity on the ictal-interictal continuum.^4,5,6^ Patients who develop epileptiform EEG activity generally have worse clinical outcomes.^5,7,8,9,10,11^ It remains controversial whether this association reflects a causal relationship mediated by secondary brain injury, as has been observed in other disease states,^12,13,14^ or if abnormal EEG findings are simply a marker of primary injury severity.^15^ Regardless, most clinicians treat convulsive or nonconvulsive status epilepticus when detected.^16,17,18,19^ In short, EEG findings have established prognostic value, and detection of EEG abnormalities may alter clinical care. Based on these facts, consensus guidelines strongly recommend that patients who are comatose after cardiac arrest undergo frequent or continuous EEG monitoring.^20^

Because abnormal EEG activity in patients who are comatose and undergo treatment in the intensive care unit (ICU) is often transient and episodic, 48 hours or more of continuous EEG monitoring may be necessary to achieve reasonable sensitivity.^21,22,23^ Unfortunately, continuous monitoring is resource intensive and is not available in all settings. To determine the yield over time of EEG monitoring after cardiac arrest, we analyzed a large, multicenter cohort of patients who were continuously monitored after cardiac arrest to determine the time-dependent sensitivity for detecting (1) epileptiform patterns associated with patient outcome, and (2) potentially treatable seizures likely to cause secondary brain injury. We compared the sensitivity and timeliness of detection of continuous EEG with several widely available alternative strategies of brief intermittent monitoring (ie, 20 minutes or less). The objectives of this study were to determine the difference in sensitivity and delay between intermittent and continuous EEG monitoring for epileptiform event detection. We then quantified the outcomes of these differences by incorporating continuous or brief intermittent EEG results into models predicting neurological outcome based in multimodality neurological evaluation. This approach tested the hypothesis that brief intermittent EEG provides less prognostic information than continuous EEG.

## Methods

### Patients and Setting

We performed a multicenter observational cohort study of consecutive patients, admitted to 2 academic medical centers, who were comatose following cardiac arrest and underwent continuous EEG monitoring from September 2010 to January 2018. During the study period, it was standard of care at both institutions to monitor these patients with continuous EEG, except in cases with rapid awakening prior to monitoring initiation; early limitations of care because of prior advanced directives; refractory multisystem organ failure or rearrest; or nonsurvivable cerebral edema on initial brain imaging. We excluded patients who experienced cardiac arrest as a result of trauma or neurological catastrophe. To minimize bias, we further excluded patients who underwent fewer than 24 hours of monitoring and patients with interruptions in monitoring totaling more than 10% of the overall observation period. We identified patients from prospective registries separately maintained by each center. The University of Pittsburgh Human Research Protection Office approved all aspects of this study with a waiver of informed consent as minimal risk research. We followed the reporting requirements of the Strengthening the Reporting of Observational Studies in Epidemiology (STROBE) statement.^24^

UPMC Presbyterian Hospital is a 798-bed tertiary care academic medical center, and it receives a high volume of patients after cardiac arrest via interfacility transfer.^25^ Patients at this center are cared for by members of the University of Pittsburgh Post–Cardiac Arrest Service, the structure and function of which have been previously described.^25,26^ Patients with potentially treatable epileptiform EEG activity (defined in the subsection EEG-Related Variables) receive antiepileptic drug (AED) treatment in a standardized fashion.^6,27^ The hospital has full-time in-house EEG technologists, and EEG monitoring is typically initiated on ICU arrival, an average of 6 to 8 hours after return of spontaneous circulation.^27^ We use 22 gold-plated cup electrodes placed in the standard International 10–20 system and record data using XLTech Natus Neuroworks digital video/EEG systems (Natus Medical Inc). We typically continue EEG monitoring until awakening, death, or approximately 48 hours of data are acquired without any findings considered actionable by the treating team.

The University of Alabama at Birmingham (UAB) Hospital is a 1157-bed tertiary care academic medical center. Patients treated at this center are cared for by members of the UAB Therapeutic Hypothermia Team, a 6-physician consult service providing standardized, goal-directed care for consecutive patients who experience out-of-hospital cardiac arrest according to current American Heart Association guidelines modeled after the University of Pittsburgh Post–Cardiac Arrest Service. When activated, these physicians mobilize a multidisciplinary team equipped to provide a bundle of care, including 24 hours of targeted temperature management to 33 °C, early percutaneous coronary intervention, ventilator management, continuous EEG, neurocognitive and electrophysiology evaluation, and extracorporal cardiopulmonary resuscitation or other mechanical circulatory support. UAB is staffed by EEG technologists who are available at all hours, and EEG monitoring is typically initiated within 3 hours of arrival in the emergency department or ICU (if direct transfer). Acquisition, recording, and interpretation of EEG activity is similar to methods used in the Pittsburgh cohort, except that, in cases where prolonged periods of EEG monitoring yield invariant results, the interpreting attending physician may archive only representative clips instead of the entire recording.

### EEG-Related Variables

Two study coauthors (P.J.C. and M.E.B.) jointly annotated EEG recordings. We defined EEG states over time using an adaptation of the American Clinical Neurophysiology Society guidelines,^28^ as previously described,^6^ with specific attention to patterns identified as particularly common or important in prior post–cardiac arrest research.^4,6,27,28,29,30^ We categorized background activity as suppression (less than 10 µV), suppression burst (also referred to as *burst suppression* or *discontinuous*), or continuous (including continuous with periods of attenuation [ie, nearly continuous]). We separately categorized superimposed epileptiform activity as no activity (ie, suppression); nonepileptiform activity; nonperiodic epileptiform discharges; periodic epileptiform discharges (including generalized, lateralized, bilateral independent, and other periodic discharges); polyspike wave discharges, regardless of presence or absences of associated myoclonus; and electrographic seizures (including both evolving seizures and periodic discharges greater than 2.5 Hz).

Not all EEG patterns on the ictal-interictal continuum are believed to have the same prognostic significance, potential to cause secondary brain injury, or responsiveness to treatment with AEDs.^6,11,31^ For example, occasional epileptiform discharges scattered throughout an otherwise normal recording are likely both less ominous for prognosis and less injurious than electrographic status epilepticus. Some patterns, such as burst suppression with identical bursts, are unique to global anoxic injury—they are important for prognosis but are more likely epiphenomena of devastating injury than treatable mediators of additional injury.^10,29^ For the purposes of this analysis, we categorized EEG patterns as risk factors (ie, less likely to be prognostically or clinically important but instead reflective of epileptiform potential) or events (ie, strongly associated with outcome or likely to cause secondary brain injury). We tested 2 sets of event definitions—1 designed to detect EEG findings associated with outcome at hospital discharge (*prognostic events*) and 1 designed to detect potentially treatment responsive classical seizures likely to cause secondary brain injury (*potentially treatable seizures*) (Table 1). These definitions were selected a priori based on prior studies specific to the cardiac arrest population.^4,6,10,16,18,19,27,28,29,30,34^

**Table 1.** Definitions of Risk States and Clinical Events

Definition	Clinical event(s)	Risk state	Rationale
Prognostic events	Electrographic seizures or periodic discharges >2.5 Hz, regardless of interictal background activity^8,11,32,33^; Polyspike wave or other epileptiform discharges on an otherwise suppressed background, regardless of associated myoclonus, including burst suppression with identical bursts^8,10,11^	Nonperiodic epileptiform discharges or periodic discharges ≤2.5 Hz with at least some background activity	Optimized to detect prognostically important epileptiform events
Potentially treatable seizures	Electrographic seizures and status epilepticus with continuous interictal background activity^6,16,19,34^; Periodic discharges >2.5 Hz with continuous background activity^6,12,16,19,34^	All other epileptiform activity	Optimized to detect potentially treatable seizures likely to cause secondary brain injury

### Baseline Clinical Variables

From each center’s prospective registry, we extracted standard demographic and clinical variables, including patient age, sex, cardiac arrest location (out of hospital vs in hospital), initial arrest rhythm (dichotomized as shockable [ventricular tachycardia or fibrillation] vs nonshockable [pulseless electrical activity or asystole]), duration from collapse to return of spontaneous circulation, receipt of cardiac catheterization, and use of targeted temperature management. In the subset of patients who experienced an out-of-hospital cardiac arrest, we additionally abstracted witnessed status and receipt of layperson cardiopulmonary resuscitation. For the Pittsburgh cohort only, we further abstracted Pittsburgh Cardiac Arrest Category, a validated measure of post–cardiac arrest illness determined from initial neurological examination and severity of cardiopulmonary failure^2^; gray matter to white matter Hounsfield unit ratio on admission brain computerized tomography (an estimate of cerebral edema severity)^35^; and etiology of cardiac arrest, adjudicated as previously described.^36^ These final 3 variables were not available in the Alabama registry.

### Patient Outcomes

From each registry, we abstracted 3 patient outcomes: survival to hospital discharge, awakening from coma (defined as following verbal commands at any point during hospitalization), and modified Rankin Scale score at hospital discharge. These outcomes were ascertained prospectively via daily assessment by an attending physician member of the Post–Cardiac Arrest Service (Pittsburgh) or Therapeutic Hypothermia Team (Alabama). In our adjusted outcome models, we considered patients who awakened and survived to hospital discharge to have a favorable outcome (nonvegetative survival) compared with those who died in the hospital or were discharged in an unconscious state.

### Time-Dependent Sensitivity of Continuous EEG

We applied a multistate survival analysis to our simplified 3-level ordinal states (nothing epileptiform; risk state; event), as has been described in detail elsewhere.^22,37^ We used an illness-death assumption that allowed transitions to occur in only 1 direction (ie, once a risk state [ie, illness] was observed, the patient could never transition back to the nonepileptiform state; once an event was observed [death], the patient could not transition back to another state).^22^ This allowed simple quantification of the probability of an EEG event being observed with continued observation out to 5 days based on the presence or absence of previously observed risk states. Prior work by our group has demonstrated that continuity of the initial EEG background activity is associated with both AED responsiveness and incidence of epileptiform events after cardiac arrest.^6^ Therefore, we also considered baseline EEG risk state (generalized suppression, burst suppression, continuous or near-continuous activity) in addition to time-varying (ie, EEG-based) risk states to stratify our results.

### Statistical Analysis

We compared continuous EEG monitoring (our reference criterion standard) to several alternative strategies. First, we considered the scenario in which no EEG was performed. Next, we considered 4 scenarios based on simulated spot EEG monitoring options. In the first 2 scenarios, we simulated a 40-minute spot EEG recording obtained randomly (1) within 24 hours of patient arrival and (2) during the first available daylight hours (between 8 am and 5 pm, 7 days per week). We then simulated scenarios where spot EEG was converted to continuous monitoring if a risk state were detected, again obtaining spot EEG (3) randomly within 24 hours of arrival and (4) during the first available daylight hours. For each scenario, we completed 1000 simulations and verified this number was sufficient by inspecting Monte Carlo errors.^38^ We calculated the sensitivity for event detection in each scenario at the level of the patient, then calculated delay-to-event detection (restricted to the subgroup of patients in whom an event was observed both on continuous EEG and in simulation). Then, we used the results of each simulation to construct an adjusted logistic regression model using all available clinical data (Table 2) and predicting nonvegetative survival to hospital discharge. We compared the distribution of areas under the curve and the proportion of individuals reaching an outcome probability of less than 1% (ie, sufficiently unlikely to recover that withdrawal of life-sustaining therapy might be recommended^32^) to that of our criterion standard (continuous EEG) results using 2-tailed *t* tests. We calculated the variance of point estimates of the area under the receiver operating curve according to Hanley and McNeil.^39^ We considered *P* < .05 to be significant. Patients in the Alabama cohort were not included in these regression models because results of initial neurological examination and brain imaging were unknown. We used R version 3.6.1 (R Foundation) and the mstate package for multistate survival analysis and performed simulations and remaining analyses using STATA version 14 (StataCorp).

**Table 2.** Clinical Characteristics and Patient Outcomes, Stratified by Treating Center

Characteristic	No. (%)	
	Pittsburgh cohort (n = 584)	Alabama cohort (n = 175)
Age, mean (SD), y	57 (17)	58 (16)
Female	217 (37.2)	64 (36.6)
Out-of-hospital cardiac arrest	487 (83.4)	130 (74.3)
Shockable initial rhythm	171 (29.3)	61 (34.9)
Witnessed arrest^a^	317 (65.1)	103 (79.2)
Layperson CPR^a^	307 (63.0)	29 (22.3)
Epinephrine >1 mg	396 (67.8)	NA
Cardiac arrest duration, min		
≤10	118 (20.2)	12 (6.9)
11 to 30	275 (47.1)	24 (13.7)
>30	78 (13.4)	8 (4.6)
Unknown	113 (19.3)	131 (74.9)
Pittsburgh cardiac arrest category^b^		
II	153 (26.2)	NA
III	61 (10.4)	NA
IV	324 (55.5)	NA
Not assessable	49 (8.4)	NA
Gray–white ratio on admission brain CT		
<1.2	53 (9.1)	NA
1.2-1.4	320 (54.8)	NA
>1.4	102 (17.5)	NA
Not assessable or not done	109 (18.7)	NA
Cardiac etiology of cardiac arrest	121 (20.7)	NA
Cardiac catheterization performed	137 (23.5)	29 (16.6)
Received TTM	566 (96.9)	172 (98.3)
Survived to discharge	177 (30.3)	42 (24.0)
mRS score of 0-2 at discharge, No./total No. (%)^c^	26/177 (14.7)	13/42 (31.0)

Abbreviations: NA, not available; TTM, targeted temperature management; mRS, modified Rankin Scale.

^a^ Percentage is expressed including only out-of-hospital cardiac arrests.

^b^ Scoring of Pittsburgh Cardiac Arrest Category includes assessment of neurological examination, so it cannot be assessed in the context of neuromuscular blockade or other confounders such as refractory shock or hypoxemia.

^c^ Percentage is expressed including only survivors.

## Results

Overall, 759 participants (584 in Pittsburgh and 175 in Alabama) were included in analysis (281 [37%] female; mean [SD] age 58 [17] years). Of this cohort, 617 patients (81%) experienced out-of-hospital cardiac arrest, and a minority (31%) had an initial shockable rhythm (Table 2); 219 patients (29%) survived to hospital discharge, and 36 survivors (26%) had favorable functional recovery at discharge. A total of 47 924 hours of EEG data were analyzed (mean time per patient 63 [33] hours). Results of the 2 main multistate models are shown in the Figure. In addition, 414 patients (55%) developed epileptiform EEG activity at least once during monitoring. Using the prognostic events definition, we found that 293 patients (39%) had an EEG event and 275 (36%) developed a risk state. With the stricter potentially treatable seizure definition, 26 patients (3%) had EEG events and 411 (54%) developed a risk state. Of patients with intact interictal cortical background activity and no risk state on initial monitoring, only 2% developed seizures on subsequent monitoring. These proportions did not differ across cohorts. Results stratified by first observed EEG state are presented in Table 3 and in eFigures 1 and 2 in the Supplement. Depending on baseline state, achieving 95% sensitivity required between 0 and 51 hours of continuous monitoring for detection of prognostic events and 0 to 53 hours for detection of potentially treatable seizures.

**Figure.**
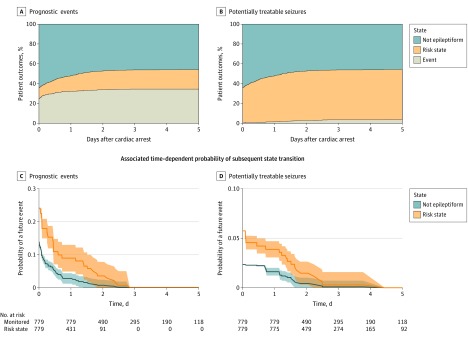
Cumulative Incidence of Risk States and Clinical Events Over Time A and B, Cumulative proportion of subjects who experience no epileptiform activity, a risk state, or an EEG event, as categorized in Table 1. C and D, Probability of detecting a future EEG event with continued monitoring among patients with no prior epileptiform activity and those previously entering a risk state.

**Table 3.** Overall Event and Seizure Probabilities and Minimum Duration of Observation Without an Event Needed to Achieve Low Probability That an Event Will Ever Occur Subsequently

Initial background	Risk state observed prior?	Overall event probability	Time to event probability below threshold, h		
			<0.1	<0.05	<0.01
**Prognostic events**					
Overall	No	0.14	2	12	43
	Yes	0.24	14	43	60
Suppressed	No	0.27	9	14	57
	Yes	0.61	14	51	60
Burst suppressed	No	0.25	13	25	37
	Yes	0.23	11	37	44
Continuous	No	0.03	0	0	39
	Yes	0.21	7	36	68
**Potentially treatable seizures**					
Overall	No	0.02	0	0	36
	Yes	0.06	0	2	57
Suppressed	No	0.05	0	0	60
	Yes	0.09	0	53	60
Burst suppressed	No	0.03	0	0	28
	Yes	0.04	0	0	39
Continuous	No	0.02	0	0	36
	Yes	0.09	0	17	56

Overall sensitivity of spot EEG monitoring strategies for event detection was low compared with continuous EEG. Using the prognostic events definition, sensitivity ranged from 66% (95% CI, 62%-69%) to 68% (95% CI, 66%-70%) for spot EEG alone and increased to 76% (95% CI, 74%-78%) to 79% (95% CI, 77%-81%) if observation of a risk state prompted conversion to continuous monitoring thereafter (Table 4). Using the potentially treatable seizure definition, sensitivity ranged from 7% (95% CI, 4%-12%) to 8% (95% CI, 4%-12%) for spot EEG alone and increased to a range of 37% (95% CI, 31%-46%) to 42% (95% CI, 38%-46%) if observation of a risk state prompted conversion to continuous monitoring thereafter. Across models, use of simulated spot EEG resulted in an 11-hour to 12-hour delay in event detection compared with continuous EEG. Compared with no EEG monitoring, the addition of continuous EEG results classified based on the prognostic events definition to a multimodality outcome model significantly increased discriminatory power and increased the proportion of patients with near-zero estimated probability of recovery from 7% (95% CI, 5%-9%) to 26% (95% CI, 22%-30%) (*P* < .001) (Table 4). Compared with continuous EEG, simulated spot EEG resulted in a statistically but not clinically significant improvement in predictive performance across models (Table 4). There was no improvement in ability to identify patients with near-zero recovery probability using the potentially treatable seizure event definitions.

**Table 4.** Results From Primary Analyses Comparing Performance Characteristics of Various EEG Monitoring Strategies

Monitoring strategy	Event detection sensitivity, %^a^	*P* value vs cEEG	Delay to event detection, mean (SD), min^a^	Multimodality outcome model AUC	*P* value vs no EEG^b^	*P* value vs cEEG^b^	Proportion with Pr(recovery) <0.01, mean (95% CI), %^c^	*P* value vs no EEG	*P* value vs cEEG
**Prognostic events**									
No EEG performed	NA	NA	NA	0.87	NA	NA	7 (5-9)	NA	NA
Continuous EEG	100 (99-100)	NA	0	0.92	<.001	NA	26 (22-30)	<.001	NA
Random spot EEG within 24 h	66 (62-69)	<.001	653 (55)	0.91	<.001	<.001	20 (16-24)	<.001	.03
Random spot EEG, 8 am to 5 pm	68 (66-70)	<.001	670 (32)	0.91	<.001	<.001	21 (17-24)	<.001	.06
Random spot EEG within 24 h converted to continuous if risk state detected	76 (74-78)	<.001	661 (49)	0.91	<.001	<.001	21 (18-25)	<.001	.12
Random spot EEG 8 am to 5 pm converted to continuous if risk state detected	79 (77-81)	<.001	676 (30)	0.91	<.001	<.001	22 (18-26)	<.001	.17
**Potentially treatable seizures**									
No EEG performed	NA	NA	NA	0.87	NA	NA	7 (5-9)	NA	NA
Continuous EEG	100 (99-100)	NA	0	0.90	<.001	NA	9 (7-12)	0.18	NA
Random spot EEG within 24 h	7 (4-12)	<.001	637 (355)	0.90	<.001	<.001	8 (6-11)	.42	.57
Random spot EEG, 8 am to 5 pm	8 (4-12)	<.001	734 (187)	0.90	<.001	<.01	9 (6-11)	.28	.45
Random spot EEG within 24 h converted to continuous if risk state detected	37 (31-46)	<.001	716 (216)	0.90	<.001	<.001	8 (6-11)	.36	.51
Random spot EEG 8 am to 5 pm converted to continuous if risk state detected	42 (38-46)	<.001	676 (30)	0.90	<.001	<.001	9 (6-11)	.32	.49

Abbreviations: AUC, area under the receiver operating curve; cEEG, continuous electroencephalography; EEG, electroencephalography; NA, not applicable; Pr, probability.

^a^ Event sensitivity and delay to detection are based on definitions from Table 1. Outcome model results (AUC and proportion with predicted recovery probability less than 1%) are derived from adjusted models predicting nonvegetative survival to discharge based on clinical characteristics and multimodality assessment of neurological injury with or without various EEG findings included. Delay to event detection is presented as means of the median values of individual simulations, where median calculations were restricted to the subset of patients with true positive events detected, and median with interquartile range for criterion standard results.

^b^ *P* values for AUC are calculated with *t* tests using the mean and SD of the point estimate AUC or distribution of simulation results, as appropriate. The variance of point estimates of AUC were calculated according to Hanley and McNeil.^39^

^c^ 95% confidence intervals are calculated as Agresti-Coull approximations of the binomial confidence interval for point estimates and determined from the distribution of bootstrapped point estimates for simulation results.

## Discussion

We present results from a large cohort of EEG-monitored patients who were comatose and resuscitated from cardiac arrest. More than half of patients had epileptiform EEG activity observed during continuous monitoring, despite the fact that monitoring was performed routinely rather than selectively based on clinical suspicion. Prior reports of EEG findings after cardiac arrest use variable definitions to define epileptiform events or focus on specific types of EEG activity, making direct comparison difficult, but the frequency of observed epileptiform activity in our cohort is likely somewhat higher than previously described.^8,18,33,40,41^ Overall severity of anoxic brain injury in our cohort was also greater than these studies. The most common initial neurological examination in the Pittsburgh cohort was coma with loss of some brainstem reflexes, and unfavorable arrest characteristics were considerably more common than reported in other studies (eg, fewer than one-third of patients presented with a shockable initial rhythm, most arrests were not due to cardiac etiology). This may be because of regional or cultural variation in decisions to attempt resuscitation, transport to the hospital, or offer aggressive care on ICU admission or may reflect local differences in cardiac arrest epidemiology. Regardless, that this cohort with severe brain injuries had a high frequency of epileptiform activity was not unexpected. By contrast, frank electrographic seizures with intact interictal cortical background activity, a finding likely to cause secondary brain injury and respond to treatment, were rare.^6^ Compared with general and neuroscience ICU populations, where up to 1 in 5 patients who are comatose is found to have nonconvulsive seizures,^21,42,43,44^ cumulative incidence in our population after 5 days of monitoring was only 3%.

In the general ICU population, early work by Claassen et al^21^ suggests that sensitivity for seizure detection exceeds 90% only after 24 to 48 hours of continuous EEG monitoring. Subsequent studies demonstrate clinical characteristics on the ictal-interictal spectrum, such as presence of coma or prior history of seizures and observation of epileptiform transients, all increase an individual’s risk of developing seizures, and thus affect the time-dependent probability of seizure detection.^22,23^ Our findings confirm that short durations of EEG monitoring are insensitive for detection of epileptiform activity. This is particularly true for detection of potentially treatable seizures, where our simulations demonstrate that intermittent monitoring is only 7% to 8% sensitive and would delay detection and initiation of treatment by many hours. Sensitivity for detection of prognostic epileptiform findings is considerably higher, probably because many of these findings are persistent rather than occurring transiently.^29,41^ Still, spot EEG monitoring detected only 66% to 68% of these events, somewhat lower than a 2013 report,^45^ with a mean delay of more than 10 hours in event detection compared with continuous monitoring.

Across patients, 48 hours of observation achieved 95% sensitivity for detection for prognostic EEG events. Despite being insensitive for detection of prognostic epileptiform events, simulated spot EEGs performed similarly to continuous monitoring when added to adjusted outcome models, and all EEG monitoring strategies performed better than prognostication in the absence of EEG. These observations are consistent with results of prior smaller studies^9,46^ and suggest that missed epileptiform activity in these patients conveys information that is largely collinear with other prognostic modalities. Insofar as modest delays before prognostic data become available are acceptable and seizure detection is deemed unimportant, brief intermittent EEG monitoring may thus be an economically favorable alternative to continuous monitoring with similar prognostic performance. A notable subgroup are those patients with continuous cortical background activity and no epileptiform activity on initial monitoring. Among this subgroup, the probability of observing prognostic or clinically important epileptiform activity on subsequent monitoring is extremely low (2%, [Table 3]). These patients seem unlikely to derive benefit from conversion of a spot EEG recording to continuous monitoring.

### Limitations

Our study has several important limitations. Although we were able to calculate time-dependent sensitivities and perform simulations for the full multicenter cohort, prognostic data for the Alabama cohort were limited, and these patients were not included in outcome modeling. Moreover, the composition of our cohort may not reflect patterns seen outside the US (eg, the high proportion of patients with nonshockable initial rhythms). The generalizability of this component of our results is therefore lessened. Detailed, time-varying medication and other treatment characteristics were also not available for all patients, so we are unable to report the influence of sedation, temperature management, and other factors on time to detection of first event. We have previously reported that AED treatment and hypothermia are not associated with the probability of developing many epileptiform patterns after cardiac arrest, although they are associated with a reduced probability of the seizures detected using potentially treatable seizure event definitions.^6^ The influence of these unmeasured confounders on results from this model in particular are unknown but potentially important. Our classification of EEG states and events is also imperfect. Quantitative EEG analysis may yield additional prognostic information,^47^ as do characteristics such as reactivity.^48^ Thus, continuous EEG may still add prognostic information to multimodality outcome models when features we did not evaluate are included as predictors. Additionally, because discontinuation of EEG monitoring was not rigidly standardized, it is possible that this was an imperfect criterion standard for epileptiform event detection. To minimize this bias, we excluded patients monitored less than 24 hours as well as those with substantial missing data. Nevertheless, decreasing time-dependent sensitivity after 24 hours is influenced in part by the termination of monitoring.

### Conclusions

We found that compared with continuous EEG monitoring, brief intermittent monitoring is insensitive and slow for detection of epileptiform events observed in most of this cohort with severe brain injuries. Both intermittent and continuous EEG significantly improved multimodality outcome prediction, but continuous monitoring appeared to add little information compared with brief intermittent monitoring.

## Notes

Supplement. **eFigure 1.** Time-Dependent Probabilities of Observing a First EEG Event if Monitoring Were Continued, Stratified by Initial EEG Background Activity (1a – Suppressed Background; 1b – Burst Suppression; 1c – Continuous) and Whether or Not a Risk State Was Previously Observed Using Prognostic Event Definitions **eFigure 2.** Time-Dependent Probabilities of Observing a First EEG Event if Monitoring Were Continued, Stratified by Initial EEG Background Activity (2a – Suppressed Background; 2b – Burst Suppression; 2c – Continuous) and Whether or Not a Risk State Was Previously Observed Using Potentially Treatable Seizures Definitions

## References

[1] Benjamin EJ, Virani SS, Callaway CW, ; American Heart Association Council on Epidemiology and Prevention Statistics Committee and Stroke Statistics Subcommittee Heart disease and stroke statistics—2018 update: a report from the American Heart Association. Circulation. 2018;137(12):-. doi:10.1161/CIR.0000000000000558 29386200

[2] Coppler PJ, Elmer J, Calderon L, . Post Cardiac Arrest Service Validation of the Pittsburgh Cardiac Arrest Category illness severity score. Resuscitation. 2015;89:86-92. doi:10.1016/j.resuscitation.2015.01.020 25636896 PMC4580975

[3] Rittenberger JC, Tisherman SA, Holm MB, Guyette FX, Callaway CW An early, novel illness severity score to predict outcome after cardiac arrest. Resuscitation. 2011;82(11):1399-1404. doi:10.1016/j.resuscitation.2011.06.024 21756969 PMC3196030

[4] Cloostermans MC, van Meulen FB, Eertman CJ, Hom HW, van Putten MJ Continuous electroencephalography monitoring for early prediction of neurological outcome in postanoxic patients after cardiac arrest: a prospective cohort study. Crit Care Med. 2012;40(10):2867-2875. doi:10.1097/CCM.0b013e31825b94f0 22824933

[5] Rossetti AO, Tovar Quiroga DF, Juan E, Electroencephalography predicts poor and good outcomes after cardiac arrest: a two-center study. Crit Care Med. 2017;45(7):e674-e682. doi:10.1097/CCM.0000000000002337 28406812

[6] Solanki P, Coppler PJ, Kvaløy JT, Baldwin MA, Callaway CW, Elmer J; Pittsburgh Post-Cardiac Arrest Service Association of antiepileptic drugs with resolution of epileptiform activity after cardiac arrest. Resuscitation. 2019;142:82-90. doi:10.1016/j.resuscitation.2019.07.007 31325554 PMC7286066

[7] Tjepkema-Cloostermans MC, Hofmeijer J, Trof RJ, Blans MJ, Beishuizen A, van Putten MJ Electroencephalogram predicts outcome in patients with postanoxic coma during mild therapeutic hypothermia. Crit Care Med. 2015;43(1):159-167. doi:10.1097/CCM.0000000000000626 25251761

[8] Westhall E, Rossetti AO, van Rootselaar AF, ; TTM-Trial Investigators Standardized EEG interpretation accurately predicts prognosis after cardiac arrest. Neurology. 2016;86(16):1482-1490. doi:10.1212/WNL.0000000000002462 26865516 PMC4836886

[9] Hofmeijer J, Beernink TM, Bosch FH, Beishuizen A, Tjepkema-Cloostermans MC, van Putten MJ Early EEG contributes to multimodal outcome prediction of postanoxic coma. Neurology. 2015;85(2):137-143. doi:10.1212/WNL.0000000000001742 26070341 PMC4515041

[10] Hofmeijer J, Tjepkema-Cloostermans MC, van Putten MJ Burst-suppression with identical bursts: a distinct EEG pattern with poor outcome in postanoxic coma. Clin Neurophysiol. 2014;125(5):947-954. doi:10.1016/j.clinph.2013.10.017 24286857

[11] Faro J, Coppler PJ, Dezfulian C, ; Pittsburgh Post-Cardiac Arrest Service Differential association of subtypes of epileptiform activity with outcome after cardiac arrest. Resuscitation. 2019;136:138-145. doi:10.1016/j.resuscitation.2018.11.022 30586605 PMC6397672

[12] Witsch J, Frey HP, Schmidt JM, Electroencephalographic periodic discharges and frequency-dependent brain tissue hypoxia in acute brain injury. JAMA Neurol. 2017;74(3):301-309. doi:10.1001/jamaneurol.2016.5325 28097330 PMC5548418

[13] Vespa P, Prins M, Ronne-Engstrom E, Increase in extracellular glutamate caused by reduced cerebral perfusion pressure and seizures after human traumatic brain injury: a microdialysis study. J Neurosurg. 1998;89(6):971-982. doi:10.3171/jns.1998.89.6.0971 9833824

[14] Claassen J, Perotte A, Albers D, Nonconvulsive seizures after subarachnoid hemorrhage: multimodal detection and outcomes. Ann Neurol. 2013;74(1):53-64. doi:10.1002/ana.23859 23813945 PMC3775941

[15] Hofmeijer J, van Putten MJ EEG in postanoxic coma: prognostic and diagnostic value. Clin Neurophysiol. 2016;127(4):2047-2055. doi:10.1016/j.clinph.2016.02.002 26971488

[16] Beretta S, Coppo A, Bianchi E, Neurologic outcome of postanoxic refractory status epilepticus after aggressive treatment. Neurology. 2018;91(23):e2153-e2162. doi:10.1212/WNL.0000000000006615 30381366

[17] Reynolds AS, Claassen J Treatment of seizures and postanoxic status epilepticus. Semin Neurol. 2017;37(1):33-39. doi:10.1055/s-0036-1593862 28147416

[18] Lybeck A, Friberg H, Aneman A, ; TTM-trial Investigators Prognostic significance of clinical seizures after cardiac arrest and target temperature management. Resuscitation. 2017;114:146-151. doi:10.1016/j.resuscitation.2017.01.017 28163232

[19] Ruijter BJ, van Putten MJ, Horn J, ; TELSTAR study group Treatment of Electroencephalographic Status Epilepticus After Cardiopulmonary Resuscitation (TELSTAR): study protocol for a randomized controlled trial. Trials. 2014;15:433. doi:10.1186/1745-6215-15-433 25377067 PMC4237766

[20] Callaway CW, Donnino MW, Fink EL, Part 8: post-cardiac arrest care: 2015 American Heart Association guidelines update for cardiopulmonary resuscitation and emergency cardiovascular care. Circulation. 2015;132(18)(suppl 2):S465-S482. doi:10.1161/CIR.0000000000000262 26472996 PMC4959439

[21] Claassen J, Mayer SA, Kowalski RG, Emerson RG, Hirsch LJ Detection of electrographic seizures with continuous EEG monitoring in critically ill patients. Neurology. 2004;62(10):1743-1748. doi:10.1212/01.WNL.0000125184.88797.62 15159471

[22] Struck AF, Osman G, Rampal N, Time-dependent risk of seizures in critically ill patients on continuous electroencephalogram. Ann Neurol. 2017;82(2):177-185. doi:10.1002/ana.24985 28681492 PMC5842678

[23] Westover MB, Shafi MM, Bianchi MT, The probability of seizures during EEG monitoring in critically ill adults. Clin Neurophysiol. 2015;126(3):463-471. doi:10.1016/j.clinph.2014.05.037 25082090 PMC4289643

[24] von Elm E, Altman DG, Egger M, Pocock SJ, Gøtzsche PC, Vandenbroucke JP; STROBE Initiative The Strengthening the Reporting of Observational Studies in Epidemiology (STROBE) statement: guidelines for reporting observational studies. Lancet. 2007;370(9596):1453-1457. doi:10.1016/S0140-6736(07)61602-X 18064739

[25] Elmer J, Rittenberger JC, Coppler PJ, Guyette FX, Doshi AA, Callaway CW; Pittsburgh Post-Cardiac Arrest Service Long-term survival benefit from treatment at a specialty center after cardiac arrest. Resuscitation. 2016;108:48-53. doi:10.1016/j.resuscitation.2016.09.008 27650862 PMC5850957

[26] Rittenberger JC, Guyette FX, Tisherman SA, DeVita MA, Alvarez RJ, Callaway CW Outcomes of a hospital-wide plan to improve care of comatose survivors of cardiac arrest. Resuscitation. 2008;79(2):198-204. doi:10.1016/j.resuscitation.2008.08.014 18951113 PMC2590640

[27] Elmer J, Gianakas JJ, Rittenberger JC, ; Pittsburgh Post-Cardiac Arrest Service Group-based trajectory modeling of suppression ratio after cardiac arrest. Neurocrit Care. 2016;25(3):415-423. doi:10.1007/s12028-016-0263-9 27033709 PMC5045751

[28] Hirsch LJ, LaRoche SM, Gaspard N, American Clinical Neurophysiology Society’s standardized critical care EEG terminology: 2012 version. J Clin Neurophysiol. 2013;30(1):1-27. doi:10.1097/WNP.0b013e3182784729 23377439

[29] Elmer J, Rittenberger JC, Faro J, ; Pittsburgh Post-Cardiac Arrest Service Clinically distinct electroencephalographic phenotypes of early myoclonus after cardiac arrest. Ann Neurol. 2016;80(2):175-184. doi:10.1002/ana.24697 27351833 PMC4982787

[30] Oh SH, Park KN, Kim YM, The prognostic value of continuous amplitude-integrated electroencephalogram applied immediately after return of spontaneous circulation in therapeutic hypothermia-treated cardiac arrest patients. Resuscitation. 2013;84(2):200-205. doi:10.1016/j.resuscitation.2012.09.031 23032690

[31] Chong DJ, Hirsch LJ Which EEG patterns warrant treatment in the critically ill? reviewing the evidence for treatment of periodic epileptiform discharges and related patterns. J Clin Neurophysiol. 2005;22(2):79-91. doi:10.1097/01.WNP.0000158699.78529.AF 15805807

[32] Sandroni C, Cariou A, Cavallaro F, Prognostication in comatose survivors of cardiac arrest: an advisory statement from the European Resuscitation Council and the European Society of Intensive Care Medicine. Intensive Care Med. 2014;40(12):1816-1831. doi:10.1007/s00134-014-3470-x 25398304 PMC4239787

[33] Rossetti AO, Logroscino G, Liaudet L, Status epilepticus: an independent outcome predictor after cerebral anoxia. Neurology. 2007;69(3):255-260. doi:10.1212/01.wnl.0000265819.36639.e0 17636063

[34] Rossetti AO, Oddo M, Liaudet L, Kaplan PW Predictors of awakening from postanoxic status epilepticus after therapeutic hypothermia. Neurology. 2009;72(8):744-749. doi:10.1212/01.wnl.0000343006.60851.62 19237704

[35] Metter RB, Rittenberger JC, Guyette FX, Callaway CW Association between a quantitative CT scan measure of brain edema and outcome after cardiac arrest. Resuscitation. 2011;82(9):1180-1185. doi:10.1016/j.resuscitation.2011.04.001 21592642 PMC3244968

[36] Chen N, Callaway CW, Guyette FX, ; Pittsburgh Post-Cardiac Arrest Service Arrest etiology among patients resuscitated from cardiac arrest. Resuscitation. 2018;130:33-40. doi:10.1016/j.resuscitation.2018.06.024 29940296 PMC6092216

[37] Putter H, Fiocco M, Geskus RB Tutorial in biostatistics: competing risks and multi-state models. Stat Med. 2007;26(11):2389-2430. doi:10.1002/sim.2712 17031868

[38] Koehler E, Brown E, Haneuse SJ On the assessment of Monte Carlo error in simulation-based statistical analyses. Am Stat. 2009;63(2):155-162. doi:10.1198/tast.2009.0030 22544972 PMC3337209

[39] Hanley JA, McNeil BJ The meaning and use of the area under a receiver operating characteristic (ROC) curve. Radiology. 1982;143(1):29-36. doi:10.1148/radiology.143.1.7063747 7063747

[40] Rittenberger JC, Popescu A, Brenner RP, Guyette FX, Callaway CW Frequency and timing of nonconvulsive status epilepticus in comatose post-cardiac arrest subjects treated with hypothermia. Neurocrit Care. 2012;16(1):114-122. doi:10.1007/s12028-011-9565-0 21638118 PMC3188346

[41] Ruijter BJ, Tjepkema-Cloostermans MC, Tromp SC, Early electroencephalography for outcome prediction of postanoxic coma: a prospective cohort study. Ann Neurol. 2019;86(2):203-214. doi:10.1002/ana.25518 31155751 PMC6771891

[42] Laccheo I, Sonmezturk H, Bhatt AB, Non-convulsive status epilepticus and non-convulsive seizures in neurological ICU patients. Neurocrit Care. 2015;22(2):202-211. doi:10.1007/s12028-014-0070-0 25246236

[43] Towne AR, Waterhouse EJ, Boggs JG, Prevalence of nonconvulsive status epilepticus in comatose patients. Neurology. 2000;54(2):340-345. doi:10.1212/WNL.54.2.340 10668693

[44] Oddo M, Carrera E, Claassen J, Mayer SA, Hirsch LJ Continuous electroencephalography in the medical intensive care unit. Crit Care Med. 2009;37(6):2051-2056. doi:10.1097/CCM.0b013e3181a00604 19384197

[45] Alvarez V, Sierra-Marcos A, Oddo M, Rossetti AO Yield of intermittent versus continuous EEG in comatose survivors of cardiac arrest treated with hypothermia. Crit Care. 2013;17(5):R190. doi:10.1186/cc12879 24007625 PMC4056115

[46] Fatuzzo D, Beuchat I, Alvarez V, Novy J, Oddo M, Rossetti AO Does continuous EEG influence prognosis in patients after cardiac arrest? Resuscitation. 2018;132:29-32. doi:10.1016/j.resuscitation.2018.08.023 30153468

[47] Nagaraj SB, Tjepkema-Cloostermans MC, Ruijter BJ, Hofmeijer J, van Putten MJAM The revised Cerebral Recovery Index improves predictions of neurological outcome after cardiac arrest. Clin Neurophysiol. 2018;129(12):2557-2566. doi:10.1016/j.clinph.2018.10.004 30390546

[48] Admiraal MM, van Rootselaar AF, Hofmeijer J, Electroencephalographic reactivity as predictor of neurological outcome in postanoxic coma: a multicenter prospective cohort study. Ann Neurol. 2019;86(1):17-27. doi:10.1002/ana.25507 31124174 PMC6618107

