# Supplementary material for: Sensitivity of Continuous Electroencephalography to Detect Ictal Activity After Cardiac Arrest
Source: JAMA Netw Open. 2020 Apr 28;3(4):e203751. doi: 10.1001/jamanetworkopen.2020.3751 (PMC7189220; doi:10.1001/jamanetworkopen.2020.3751)
Supplement: Supplement. — eFigure 1. Time-Dependent Probabilities of Observing a First EEG Event if Monitoring Were Continued, Stratified by Initial EEG Background Activity (1a – Suppressed Background; 1b – Burst Suppression; 1c – Continuous) and Whether or Not a Risk State Was Previously Observed Using Prognostic Event Definitions eFigure 2. Time-Dependent Probabilities of Observing a First EEG Event if Monitoring Were Continued, Stratified by Initial EEG Background Activity (2a – Suppressed Background; 2b – Burst Suppression; 2c – Continuous) and Whether or Not a Risk State Was Previously Observed Using Potentially Treatable Seizures Definitions [file jamanetwopen-3-e203751-s001.pdf]

Supplementary Online Content

Elmer J, Coppler PJ, Solanki P, et al. Sensitivity of Continuous Electroencephalography to Detect Ictal Activity After Cardiac Arrest. *JAMA Netw Open*. 2020;3(4):e203751. doi:10.1001/jamanetworkopen.2020.3751

eFigure 1. Time-Dependent Probabilities of Observing a First EEG Event if Monitoring Were Continued, Stratified by Initial EEG Background Activity (1a – Suppressed Background; 1b – Burst Suppression; 1c – Continuous) and Whether or Not a Risk State Was Previously Observed Using Prognostic Event Definitions
eFigure 2. Time-Dependent Probabilities of Observing a First EEG Event if Monitoring Were Continued, Stratified by Initial EEG Background Activity (2a – Suppressed Background; 2b – Burst Suppression; 2c – Continuous) and Whether or Not a Risk State Was Previously Observed Using Potentially Treatable Seizures Definitions

This supplementary material has been provided by the authors to give readers additional information about their work.

© 2020 Elmer J et al. *JAMA Netw Open.*

eFigure 1 – Time-Dependent Probabilities of Observing a First EEG Event if Monitoring Were Continued, Stratified by Initial EEG Background Activity (1a – Suppressed Background; 1b – Burst Suppression; 1c – Continuous) and Whether or Not a Risk State Was Previously Observed Using Prognostic Event Definitions

1A)

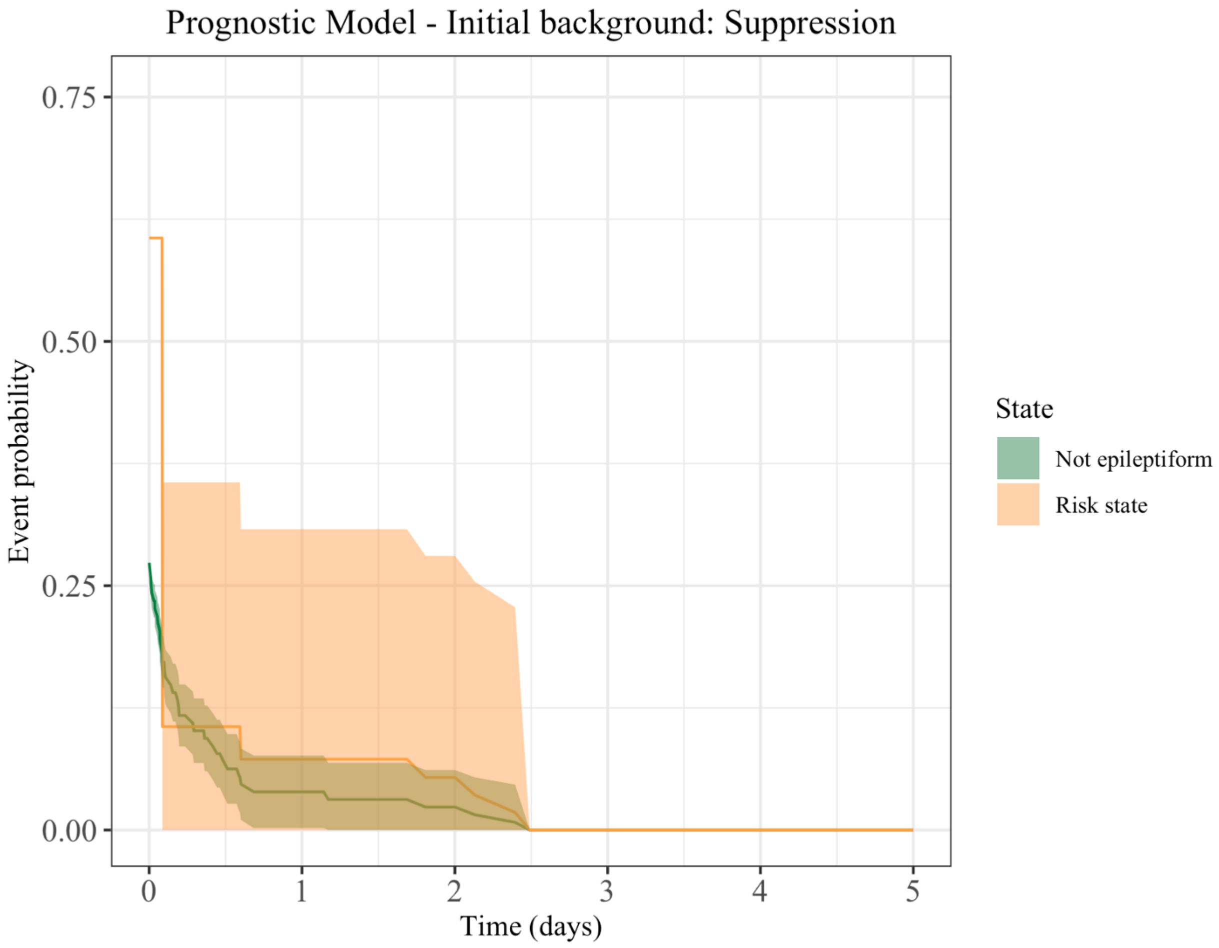

© 2020 Elmer J et al. *JAMA Netw Open.*

1B)

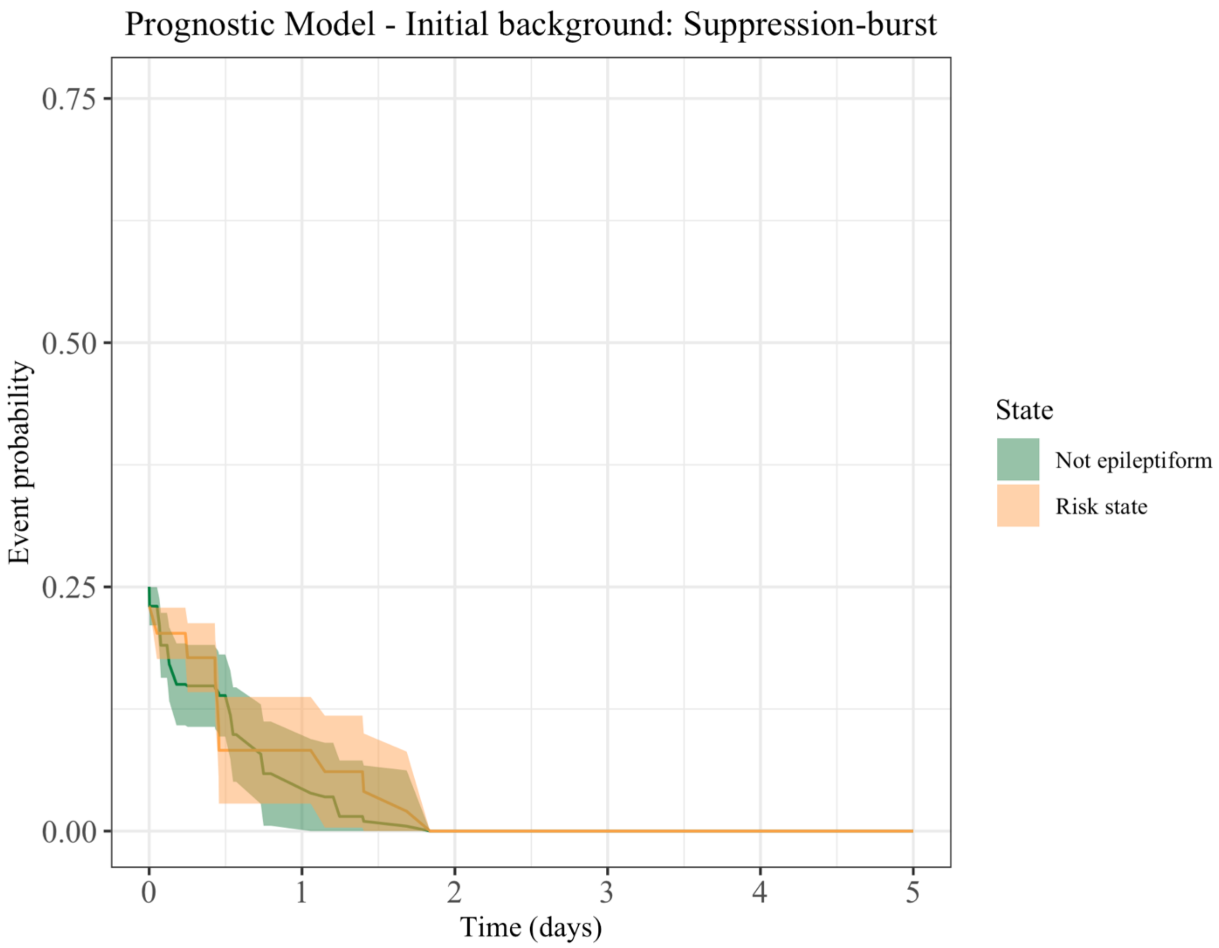

© 2020 Elmer J et al. *JAMA Netw Open.*

1C)

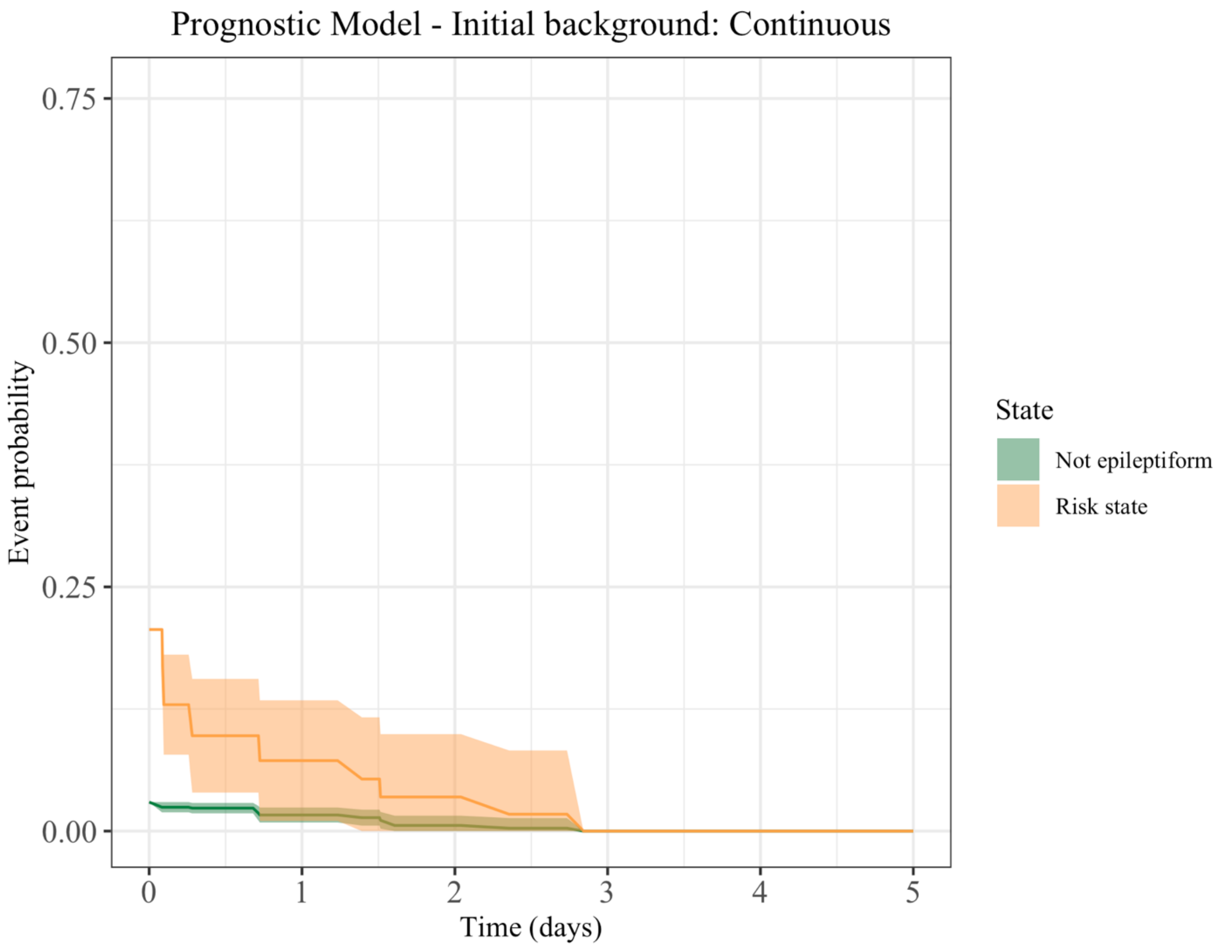

© 2020 Elmer J et al. *JAMA Netw Open.*

eFigure 2 – Time-Dependent Probabilities of Observing a First EEG Event if Monitoring Were Continued, Stratified by Initial EEG Background Activity (2a – Suppressed Background; 2b – Burst Suppression; 2c – Continuous) and Whether or Not a Risk State Was Previously Observed Using Potentially Treatable Seizures Definitions

2A)

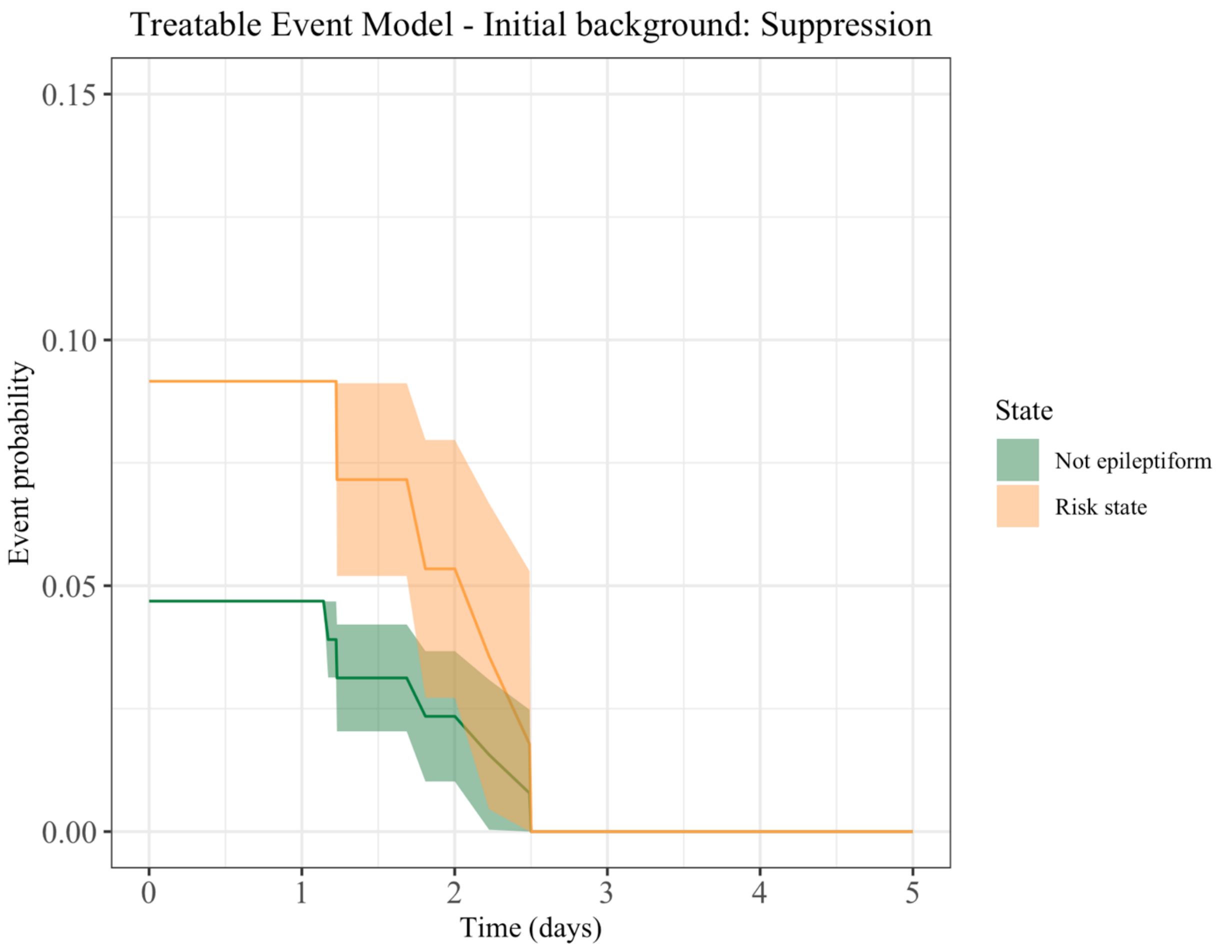

© 2020 Elmer J et al. *JAMA Netw Open.*

2B)

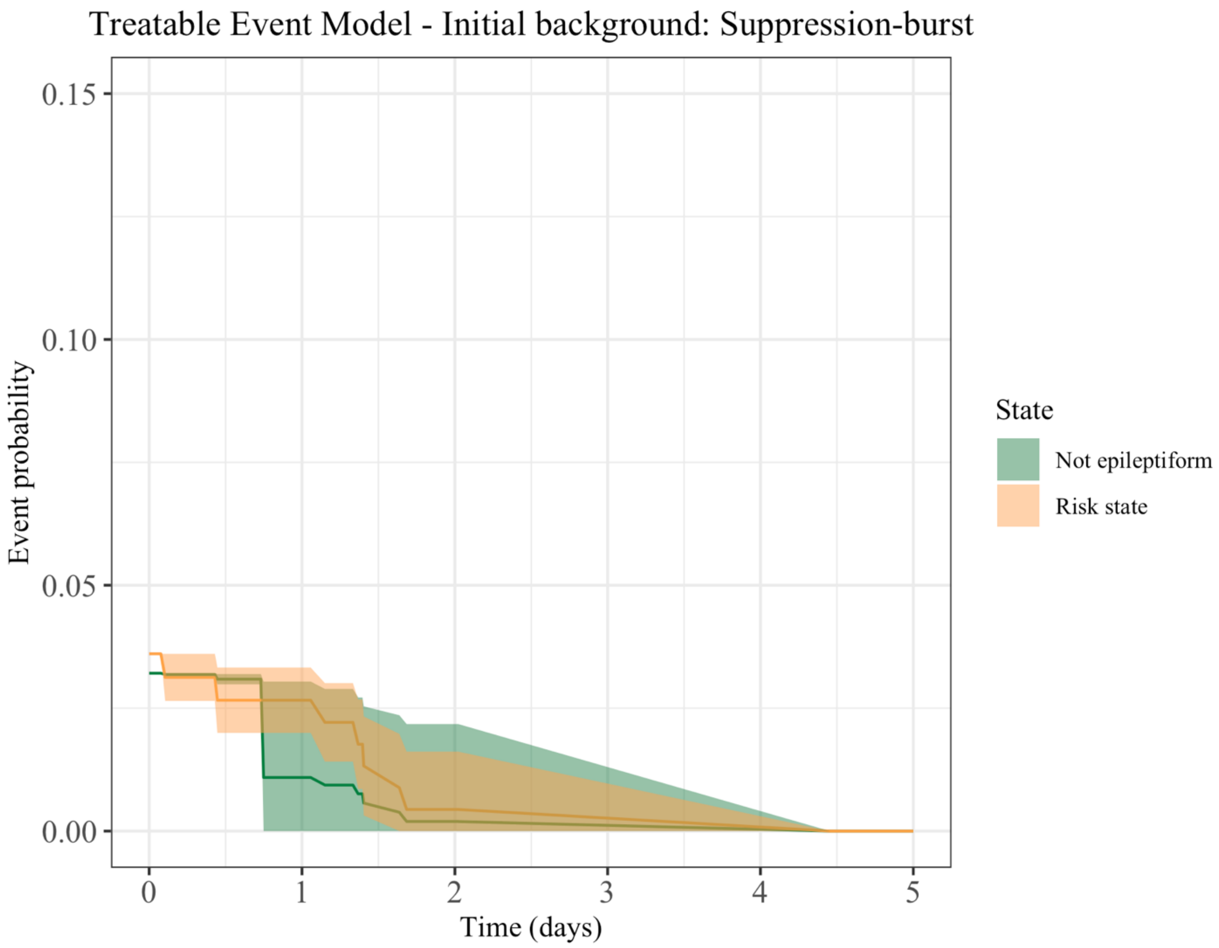

© 2020 Elmer J et al. *JAMA Netw Open.*

2C)

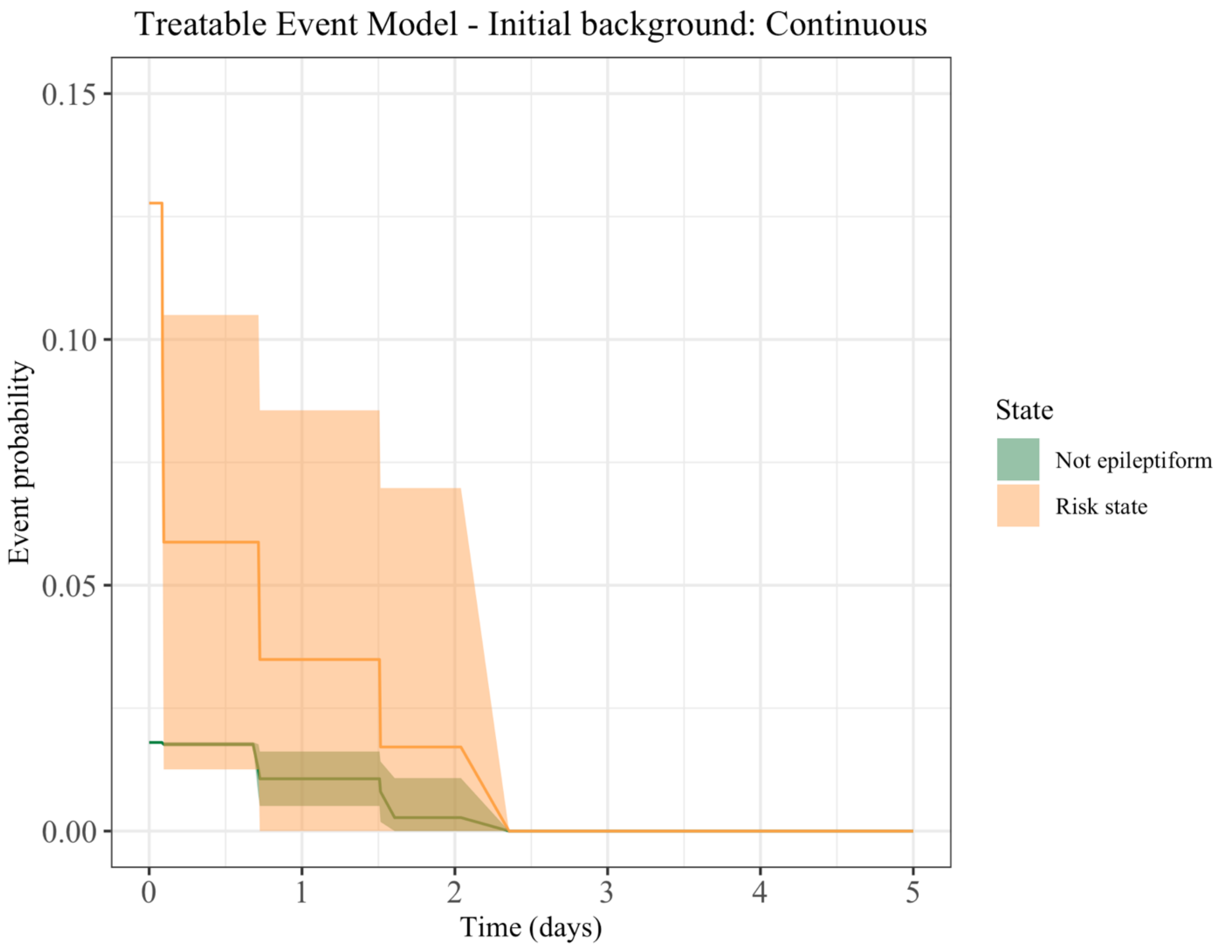

© 2020 Elmer J et al. *JAMA Netw Open.*
